# Management of a rare triad of trauma-induced pulmonary haemorrhage in an adult with Fontan physiology: a case report

**DOI:** 10.1093/ehjcr/ytag322

**Published:** 2026-05-07

**Authors:** Mukaram Rana, Marco Ochs, Lukas Probst, David M Leistner, Robert Stöhr

**Affiliations:** Department of Cardiology, Goethe University Frankfurt, University Heart Center, Theodor-Stern-Kai 7, Frankfurt 60596, Germany; Department of Cardiology, Goethe University Frankfurt, University Heart Center, Theodor-Stern-Kai 7, Frankfurt 60596, Germany; Department of Cardiology, Goethe University Frankfurt, University Heart Center, Theodor-Stern-Kai 7, Frankfurt 60596, Germany; Department of Cardiology, Goethe University Frankfurt, University Heart Center, Theodor-Stern-Kai 7, Frankfurt 60596, Germany; Institute for Cardiovascular Regeneration, Goethe University, Theodor-Stern-Kai 7, Frankfurt 60590, Germany; German Center for Cardiovascular Research, Partner Site Frankfurt Rhine-Main, Berlin 60590, Germany; Department of Cardiology, Goethe University Frankfurt, University Heart Center, Theodor-Stern-Kai 7, Frankfurt 60596, Germany

**Keywords:** Fontan circulation, VV-ECMO, Pulmonary haemorrhage, Critical care management, Case report

## Abstract

**Background:**

Patients with Fontan circulation represent a high-risk population when facing acute cardiopulmonary decompensation. The use of extracorporeal membrane oxygenation (ECMO) in this patient cohort is rare and technically demanding due to the unique circulatory physiology with passive pulmonary blood flow and predisposition to thromboembolic events.

**Case Presentation:**

We report the case of a 17-year-old male patient with a history of extracardiac Fontan palliation who developed a life-threatening diffuse pulmonary haemorrhage following blunt chest trauma. The bleeding pattern precluded both interventional and surgical management. Despite initial stabilization attempts, the patient developed progressive respiratory failure refractory to mechanical ventilation, necessitating the initiation of VV-ECMO therapy as a bridge to recovery.

Ongoing pulmonary bleeding further complicated the course, precluding the implementation of systemic anticoagulation. Consequently, a heparin-free ECMO strategy was employed. Tailored administration of coagulation factors allowed for the preservation of ECMO circuit patency while maintaining haemostasis. Following gradual respiratory improvement, the patient was successfully weaned from ECMO and discharged without residual organ dysfunction.

**Conclusion:**

This case demonstrates the need for personalized ECMO strategies, even in highly challenging haemodynamic scenarios such as Fontan physiology. Favourable outcomes can be achieved when personalized interdisciplinary management is provided in high-volume expert centres for mechanical circulation support (MCS).

Learning points

- Fontan physiology poses unique challenges for extracorporeal support due to passive pulmonary blood flow and altered haemodynamics.
- In life-threatening scenarios like pulmonary haemorrhage, anticoagulation-free VV-ECMO may be safe even in Fontan physiology, if guided by precise haemostatic monitoring.
- Multidisciplinary care in high-expertise MCS centres enables favourable outcomes through tailored ECMO, anticoagulation, and haemostatic strategies.

## Introduction

The Fontan procedure is a palliative strategy for patients with single-ventricle physiology in congenital heart disease, including double outlet right ventricle and hypoplastic left heart syndrome.^1,2^ Following staged interventions (Glenn and Norwood procedures), Fontan completion redirects systemic venous return into the pulmonary arteries, bypassing the subpulmonary ventricle and relying on passive pulmonary blood flow.^3^ Despite improved survival, Fontan patients remain vulnerable to bleeding, thromboembolic complications, and haemodynamic instability.^4^ Extracorporeal membrane oxygenation (ECMO) is rarely used in this population due to anatomical, haemostatic, and haemodynamic constraints, reserved for cardiogenic shock, low cardiac output, or respiratory failure, with high morbidity and mortality.^5,6^

## Summary figure

**Figure:**
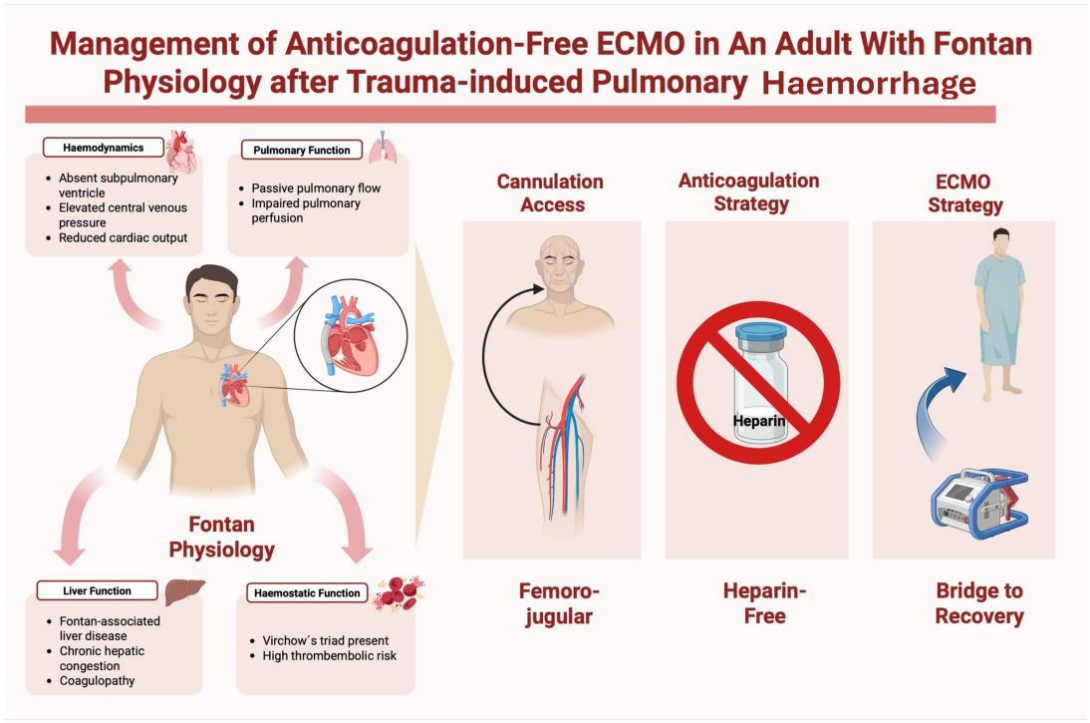
Graphical abstract illustrating the management of trauma-induced pulmonary haemorrhage in patients with Fontan physiology using anticoagulation-free mechanical circulatory support (MCS).

## Case presentation

A 17-year-old male patient presented with acute hypoxic respiratory failure following blunt thoracic trauma. His history included double outlet right ventricle, hypoplastic left ventricle, and partially anomalous pulmonary venous return; he underwent staged palliation (Norwood I-II) and Fontan completion in 2010; comorbidities included Fontan-associated liver disease. Right-heart catheterization and echocardiography showed no evidence of pulmonary arterial hypertension or shunting (no patent fenestration); recent invasive measurements confirmed a systemic arterial oxygen saturation of 99%.

Upon arrival, the patient was tachypnoeic (25/min) with an oxygen saturation of 89% on 15 L/min via a reservoir mask. Vital signs showed sinus tachycardia (112/min) and a blood pressure of 104/67 mmHg. Arterial blood gas analysis indicated respiratory alkalosis with marked hypoxaemia [pH 7.5 (7.35–7.45), PaCO_2_ 25 mmHg (35–48) mmHg, PaO_2_ 67 mmHg (83–108 mmHg)], prompting high-flow oxygen therapy.

**Figure 1:**
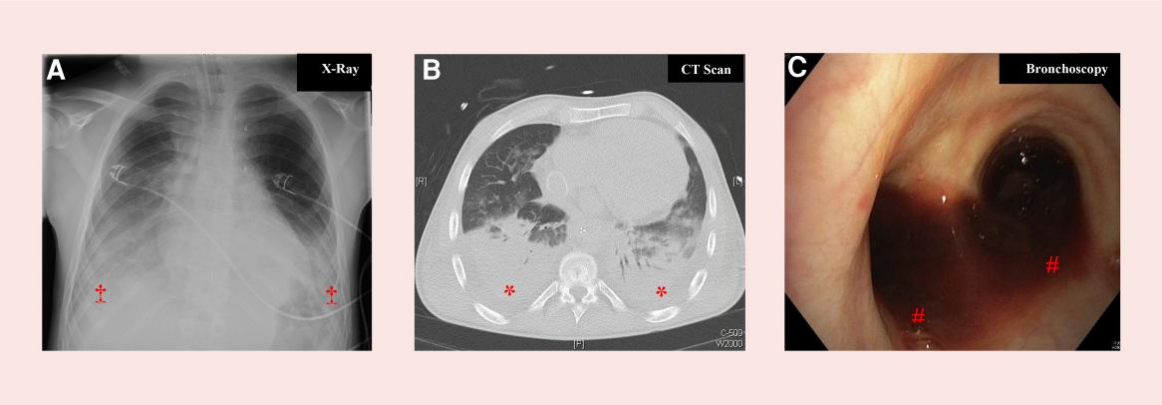
Multimodal imaging demonstrating life-threatening pulmonary haemorrhage in a patient with Fontan physiology (*A–C*). (*A*) Chest X-ray: anterior–posterior chest radiograph illustrating diffuse bilateral, predominantly lower lobe, infiltrates (‡). (*B*) Chest CT scan: cross-sectional view showing extensive bipulmonal consolidations, consistent with diffuse alveolar haemorrhage (*). (*C*) Bronchoscopy: endoscopic view of the carina (tracheal bifurcation) with evidence of bilateral pulmonary bleeding originating from both main bronchi (#).

Laboratory results indicated coagulopathy under phenprocoumon (Marcumar®) (INR: 4.04), anaemia [haemoglobin: 10.6 g/dL (13.5–17.5 g/dL)], and thrombocytopenia [platelets: 142/nl (146–328/nl)]. Chest radiography showed diffuse bilateral infiltrates without pneumothorax or pleural effusion (*Figure 1A*); computed tomography confirmed diffuse bilateral pulmonary haemorrhage without an identifiable bleeding source (*Figure 1B*). Progressive hypoxaemia refractory to non-invasive support required intubation on day 2. Initial ventilator settings were adapted to Fontan physiology [pressure-controlled ventilation (PCV), FiO_2_ 100%, PEEP 3 mbar, P_peak_ 16 mbar, respiratory rate 15/min, and inspiratory tidal volume (VT_insp_) 232 mL]. Prone positioning was considered during the early phase of respiratory deterioration. Progressive deterioration in oxygenation refractory to mechanical ventilation due to haemorrhaghe-induced intrapulmonary shunting (arterial blood gas: pH 7.3, PaO_2_ 71.9 mmHg, PaCO_2_ 40.9 mmHg on 100% inspired oxygen) and concern for haemodynamic compromise with increasing airway pressures prompted early escalation to VV-ECMO within hours of intubation (23-Fr right common femoral venous drainage and 17-Fr jugular vein return using a CardioHelp Maquet HLS 7.0 set). As severe hypoxaemia was expected to increase pulmonary vascular resistance (PVR) via hypoxemic pulmonary vasoconstriction, early pulmonary vasodilator therapy with inhaled iloprost and oral sildenafil was initiated.

**Figure 2:**
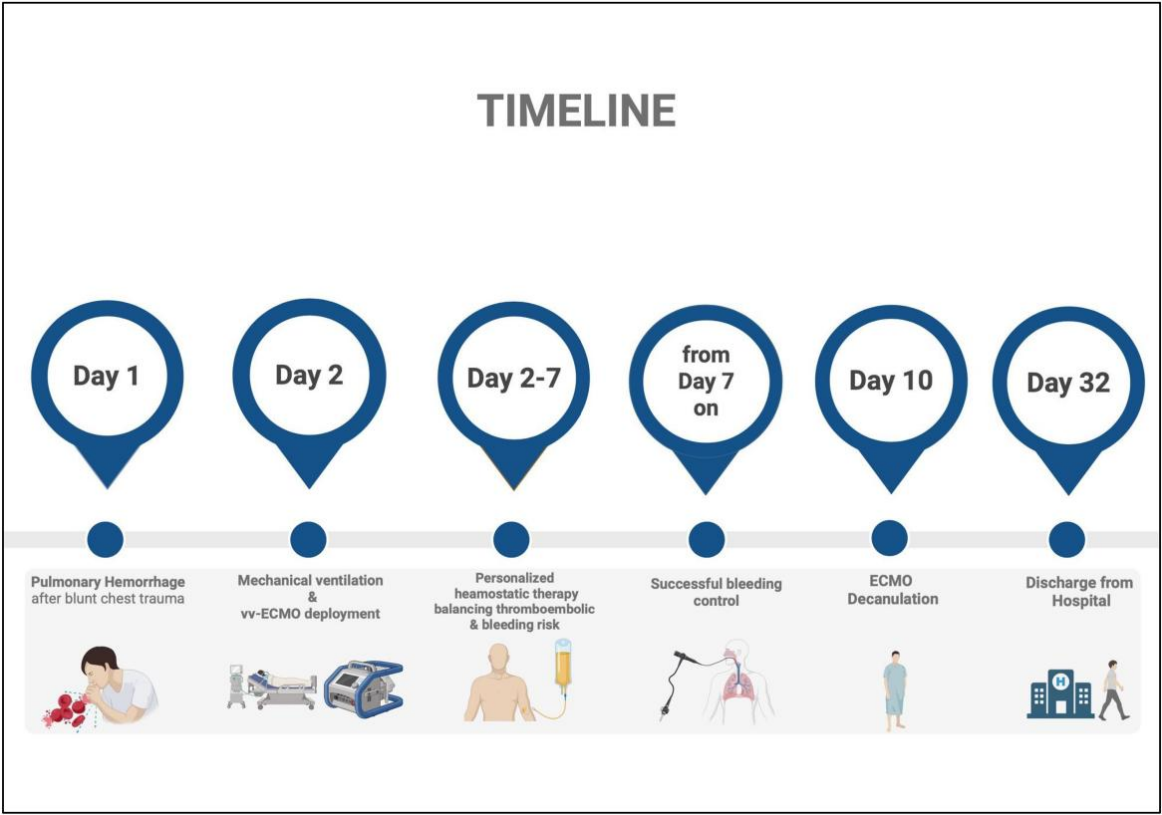
Timeline illustrating the course of hospital stay.

Bronchoscopy confirmed diffuse bleeding without a focal source (*Figure 1C*). Due to ongoing pulmonary bleeding, systemic anticoagulation was withheld, and haemostasis was supported with fibrinogen, prothrombin complex concentrate, and tranexamic acid. On day 6, reduced oxygenator capacity and hyperfibrinolysis required oxygenator replacement. Serial bronchoscopies demonstrated gradual resolution of pulmonary bleeding during ongoing ECMO support. Progressive respiratory improvement enabled successful ECMO weaning, allowing for extubation on ECMO on day 6 and ECMO explantation on day 10. During the post-ECMO course, the patient stayed haemodynamically and respiratorily stable and was discharged to a rehabilitation facility on day 32 (*Figure 2*). At the 12-month follow-up, the patient has returned to his pre-injury baseline without functional limitations or mid-term complications and continues regular outpatient follow-up.

## Discussion

This case, to our knowledge, is the first to describe the successful combination of (i) anticoagulation-free venovenous extracorporeal membrane oxygenation (VV-ECMO) as (ii) a bridge to recovery therapy in (iii) an adult with Fontan physiology presenting with trauma-induced pulmonary haemorrhage.

Despite improved survival since the Fontan procedure was first described in 1971, patients with this physiology remain vulnerable to cardiopulmonary failure due to their haemodynamic profile and a fragile balance between thromboembolic and bleeding risks.^7,8^ Fontan circulation depends on passive pulmonary blood flow, low pulmonary vascular resistance (PVR), and sufficient venous return. Severe hypoxaemia may raise PVR via pulmonary vasoconstriction (Euler–Liljestrand mechanism), and positive-pressure ventilation can reduce venous return by attenuating negative intrathoracic pressure, both reducing preload to the systemic ventricle and impairing forward flow. Therefore, we opted for pressure-controlled ventilation (PCV) and a low positive end-expiratory pressure (PEEP) strategy with early escalation to VV-ECMO to provide gas-exchange support, while allowing lung-protective ventilation without escalating airway pressures, thereby reducing the risk of compromising Fontan forward flow.

ECMO support in patients with Fontan circulation is rare and linked to high morbidity and mortality, with survival rates between 24% and 48%.^5,9^ It is predominantly reported as venoarterial (VA) support, mainly indicated in scenarios of refractory cardiogenic shock, low cardiac output syndrome, or cardiac arrest, serving as a bridge to decision or transplantation. Given predominant respiratory failure with haemodynamic stability on continuous invasive monitoring, VV-ECMO was chosen to provide extracorporeal gas exchange support, in line with guideline recommendations.^5,10^

In severe lung injury requiring VV-ECMO, several predictable challenges warrant consideration. Oxygenation may remain challenging when effective extracorporeal oxygen delivery is reduced by restricted venous drainage or relevant recirculation. In parallel, bleeding risk is elevated, especially in the presence of pulmonary haemorrhage, and requires individualized haemostatic management. Conversely, if systemic anticoagulation must be reduced or withheld, the risk of circuit thrombosis and oxygenator failure increases, potentially impairing gas exchange and necessitating close circuit surveillance and timely oxygenator exchange.

Moreover, standard ECMO cannulation strategies require modification in Fontan physiology. In paediatric cohorts, bilateral femoral access has been used but is prone to recirculation.^11^ However, in our case, femoro-jugular cannulation proved effective and safe, demonstrating the feasibility of this approach in anatomically and physiologically complex patients when managed by experienced critical care teams.

Management was further complicated by concurrent pulmonary haemorrhage and the need to maintain ECMO circuit patency in a patient with thromboembolic risk. In patients with congenital heart disease, pulmonary haemorrhage may result from aortopulmonary collaterals (APC).^12^ Standard treatment strategies involve selective embolization or surgical interventions such as lobectomy. Although APC were considered in our patient, neither previous imaging nor catheterization identified relevant collaterals. Conversely, computed tomography and bronchoscopy confirmed diffuse bilateral haemorrhage, without a focal source amenable to embolization.

Fontan patients face increased thromboembolic risk with 10-year incidence rates of 2%–25%, driven by mechanisms consistent with Virchow’s triad: flow stasis due to passive pulmonary perfusion and elevated venous pressure, endothelial dysfunction from prosthetic material and hypoxia, and hepatic coagulopathy.^13^

In our case, ECMO deployment further increased thrombosis risk while exacerbating bleeding. We therefore implemented an individualized haemostatic regimen using coagulation factor concentrates, achieving bleeding control, confirmed by serial bronchoscopies, without compromising ECMO circuit patency.

The final challenge in our case was to determine the most appropriate ECMO strategy. While ECMO support in Fontan patients is typically used as ‘bridge-to-decision’ or ‘bridge-to-transplant,’ this case highlights the potential of VV-ECMO as a successful ‘bridge-to-recovery’ in selected cases with isolated respiratory failure and preserved haemodynamics.^14^

## Conclusion

This report presents the first case of trauma-induced pulmonary haemorrhage in an adult with Fontan circulation successfully managed with anticoagulation-free VV-ECMO support. Favourable outcomes in such challenging and complex scenarios can be achieved when care is provided in specialized centres with experience in ECMO management, congenital heart disease treatment, and multidisciplinary critical care collaboration.

## Data Availability

Non-identifiable data underlying this article are available on reasonable request to the corresponding author.

## Lead author biography

Mukaram Rana, M.D. is a resident in cardiology at the University Heart Center Frankfurt, Goethe University Frankfurt, Germany. After graduating from the University of Lübeck in 2018, he has been involved in multiple research projects in the field of interventional cardiology. His current scientific and clinical areas of interest include interventional coronary therapies, intensive care medicine and advanced cardiac imaging (Coronary Computed Tomography Angiography, CCTA).

## Notes

**Consent:** Informed consent was obtained from the patient in accordance with the COPE guidelines.
